# A Highly Sensitive Biomarker of Type II Collagen C-Terminal Pro-Peptide Associated with Cartilage Formation

**DOI:** 10.3390/ijms24010454

**Published:** 2022-12-27

**Authors:** Helena Port, Anne-Christine Bay-Jensen, Yi He, Morten A. Karsdal, Thorbjørn Gantzel, Christian S. Thudium, Signe Holm Nielsen

**Affiliations:** 1 Biomarkers and Research, Nordic Bioscience, 2730 Herlev, Denmark; 2 Faculty of Health and Medical Sciences, University of Copenhagen, 2200 København, Denmark; 3 Orthopedic Surgery Unit, Gentofte University Hospital, 2820 Gentofte, Denmark

**Keywords:** C-terminal pro-peptide, type II collagen formation, cartilage, biomarker, ankylosing spondylitis, rheumatoid arthritis, osteoarthritis

## Abstract

The type II collagen C-terminal pro-peptide is one of the most abundant polypeptides in cartilage. The purpose of this study was to develop a competitive chemiluminescence enzyme-linked immunosorbent assay, CALC2, targeting this pro-peptide as a marker of cartilage formation. Technical assay parameters were evaluated. CALC2 level was measured after in vitro cleavage of recombinant type II collagen with bone morphogenetic protein-1 (BMP-1) and treatment of ex vivo human osteoarthritis (OA) cartilage explant model (HEX) with insulin-like growth factor-1 (IGF-1). Serum CALC2 levels were assessed in 18 patients with rheumatoid arthritis (RA), 19 patients with ankylosing spondylitis (AS), and 18 age- and sex-matched controls in cohort 1 and 8 patients with OA and 14 age- and sex-matched controls in cohort 2. Type II collagen cleavage with BMP-1 increased the CALC2 level. IGF-1 treatment increased the CALC2 levels in HEX compared with the untreated explants (*p* < 0.05). Results were confirmed using Western blot analysis. CALC2 levels were decreased in the patients with RA and AS compared with the healthy controls (*p* = 0.01 and *p* = 0.02, respectively). These findings indicate that CALC2 may be a novel biomarker of type II collagen formation. However, further preclinical and clinical studies are required to validate these findings.

## 1. Introduction

Type II collagen is the most abundant extracellular matrix (ECM) protein in cartilage and provides tensile elasticity and strength to the joints [1]. It is synthesised by chondrocytes, formed as a pro-collagen, and released in triple helical structures of three α-chains [2]. Each chain has pro-peptides attached at the N and C terminus, named PIINP and PIICP, respectively. PIICP is also known as chondrocalcin and is among the most highly synthesised polypeptides in cartilage [3,4]. It is cleaved off by bone morphogenetic protein-1 (BMP-1) and the associated Toll-like proteinases during maturation. It plays a critical role in the assembly of type II collagen and in the calcification of developing foetal cartilage matrix [3,5]. When cleaved off during the synthesis of type II collagen, chondrocalcin fragments are released into circulation, reflecting the true rates of type II collagen formation and cartilage metabolism [4]. Collagen turnover is part of ECM remodelling, which relies on several molecular interactions and is a crucial event in many physiological conditions. In healthy individuals, cartilage has a low collagen turnover. However, when homeostasis is disturbed, such as in rheumatic diseases, including rheumatoid arthritis (RA), ankylosis spondylitis (AS), and osteoarthritis (OA), ECM remodelling is altered, leading to an imbalance between cartilage formation and degradation [6]. Several biomarkers are already available for monitoring type II collagen degradation, such as C2M [7], CTX-II [8], and T2CM [9], and type II collagen formation, such as PRO-C2, NPII, Pcol II-C, PIIANP, PIICP, and CPII [6,10,11,12,13]. However, more studies are needed to validate these biomarkers as potential biomarkers for testing drug efficacy in anabolic treatments in pre-clinical and clinical studies.

In this study, we developed and technically evaluated an immunoassay targeting the novel epitope of chondrocalcin, named CALC2, generated from the BMP-1 cleavage at the C-terminal of the type II collagen helical domain. The potential as a translational biomarker and the biological value of CALC2 were evaluated in an ex vivo human osteoarthritis (OA) cartilage explant model (HEX). In addition, serum CALC2 levels were compared between healthy controls and patients with RA, AS, and OA. Measurement of CALC2 levels in serum samples could reflect the degree of cartilage formation and might be useful for understanding the pathogenesis of rheumatic diseases.

## 2. Results

### 2.1. CALC2 Neoepitope Identification and Monoclonal Antibody Characterisation

**Figure 1:**
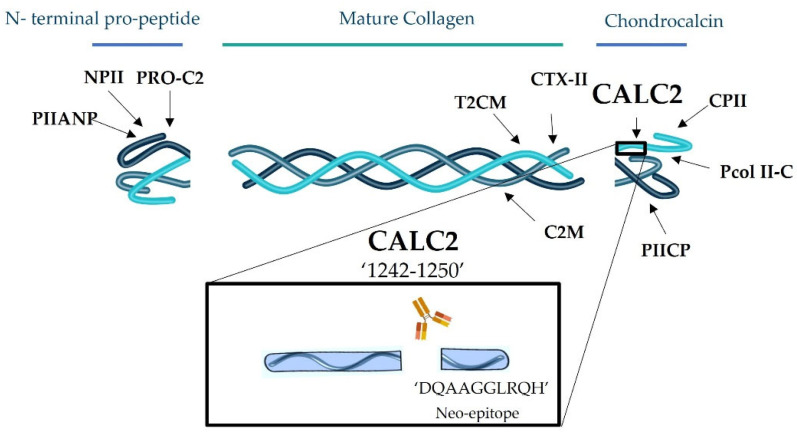
CALC2 epitope location in type II collagen. Other biomarkers of type II collagen formation (PRO-C2, NPII, PIIANP, CPII, Pcol II-C, and PIICP) and degradation (T2CM, CTX-II, and C2M) are also represented.

**Figure 2:**
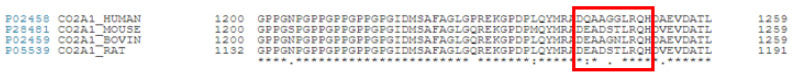
Basic Local Alignment Search Tool sequence alignment of the targeted sequence for CALC2 in human with those in mouse, bovine, and rat. The sequence is marked in red.

The epitope fragment ^1242^DQAAGGLRQH^1487^ was previously identified [3] (Figure 1). It was verified to be specific to type II collagen using the Basic Local Alignment Search Tool and did not align with mouse, bovine, and rat type II collagen sequences (Figure 2). Antibody-producing hybridoma clones were generated by fusion between mouse spleen and myeloma cells, and the monoclonal antibody with the best native reactivity, peptide affinity, and stability for the assay was identified. The antibody clone was NBH-292 # 113 4B10-2B2-1D6-1D7, and the antibody isotype was IgG1, kappa.

### 2.2. Development and Technical Evaluation of the CALC2 Assay

**Figure 3:**
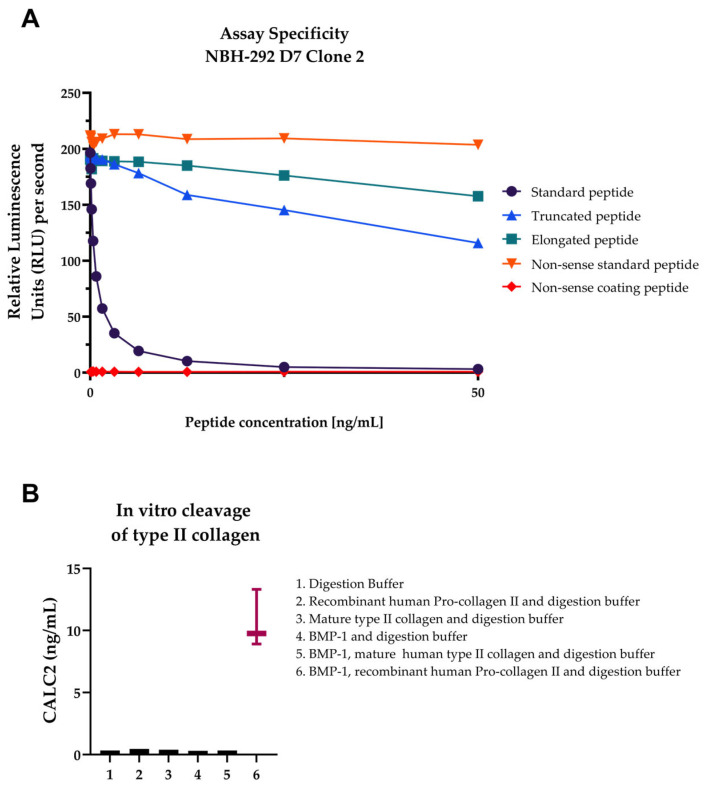
Assay validation and proof of concept. (**A**), Specificity of the monoclonal CALC2 antibody. The monoclonal antibody reactivity towards the selection, truncated, elongated, and non-sense standard and coating peptides was tested. Data are shown as B/B0, where B represents the relative light units (RLU) at given concentrations, and B0 represents the RLU at 0 ng/mL peptide, and are presented as mean values. (**B**), In vitro cleavage of recombinant type II collagen with BMP-1. Recombinant type II collagen was incubated with BMP-1 for 24 h, and CALC2 levels were quantified (*n* = 3). Data are shown as mean ± standard deviation (SD).

Assay development included the optimisation of the following parameters: buffer (pH and salt content), incubation temperature and time, coater and antibody ratio, stability, and specificity of the monoclonal antibody. A full technical validation was performed to evaluate the performance of the assay. A summary of the technical results can be found in Table 1. On the basis of 10 independent assay runs, the intra- and inter-variations were 7% and 12%, respectively, which are within the range of the acceptance criterion of <10% for the intra-assay variation and <15% for the inter-assay variation [14]. The LLOQ and ULOQ were 0.44 and 44 ng/mL, respectively. The linearity of the assay in human serum was accepted at up to twofold dilution. The spiking of the standard peptide in human serum resulted in a mean recovery of 83%. The analyte stability was acceptable for 1–5 freeze/thaw cycles and for prolonged storage of human serum samples at 4 °C and 20 °C. Neither low nor high levels of haemoglobin, lipid, and biotin interfered with the CALC2 levels in human serum. The CALC2 assay was used to test for synthetic peptides to confirm the specificity of the antibody towards the unique N-terminal site of the human target sequence ^1242^DQAAGGLRQH^1487^. No reactivity was observed towards the elongated peptide (ADQAAGGLRQH), truncated peptide (QAAGGLRQH), non-sense coating peptide (MSAFAGLGPRK-Biotin), and non-sense selection peptide (MSAFAGLGPR; Figure 3A). Higher CALC2 levels were quantified after cleavage of only recombinant type II collagen with BMP-1 (Figure 3B).

### 2.3. Biological and Clinical Evaluations of the CALC2 Assay

**Figure 4:**
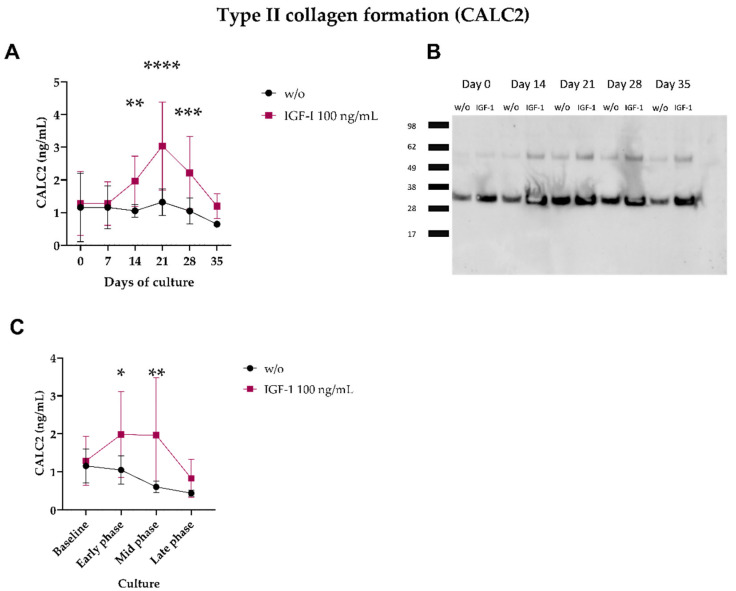
Measurements of CALC2 levels in supernatant from OA HEX cultured for 35 days in serum-free Dulbecco’s modified Eagle medium/F12 medium with insulin-like growth factor 1 (IGF-1) and a control group without (w/o) treatment. (**A**) CALC2 levels over the culture period in the first study. (**B**) Validation of the Western blot analysis results for one patient in the first study. (**C**), CALC2 levels over the culture phases in the second study, baseline (day 0), early phase (days 3, 5, and 7), mid-phase (days 10, 12, and 14), and late phase (days 17, 19, and 21). The values in panels (**A**,**C**) are shown as mean ± SD, and treatments were compared using a Mann–Whitney test at each time point. Asterisks indicate the following: * *p* < 0.05, ** *p* < 0.01, *** *p* < 0.001, **** *p* < 0.0001.

To investigate if CALC2 was released with cartilage formation, CALC2 levels were quantified in human OA explants treated with the growth factor-1 (IGF-1), which is known to induce cartilage formation by chondrocytes. In the first study, IGF-1-treated groups showed elevated CALC2 levels on days 14, 21, and 28 (*p* < 0.01, *p* < 0.0001, and *p* < 0.001, respectively; Figure 4A) compared with the untreated group, with the highest concentration on day 21. These results were further confirmed by Western blot analysis, where CALC2 detected the stronger bands on day 21 (Figure 4B). In the second study, the IGF-1-treated groups showed increased CALC2 levels in the early phase and mid-phase compared with the untreated group (*p* < 0.05 and *p* < 0.01, respectively; Figure 4C).

**Figure 5:**
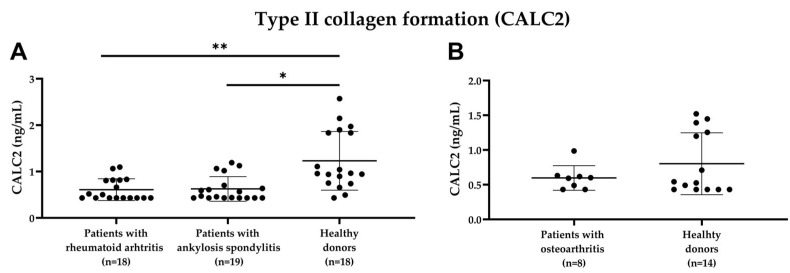
Serum CALC2 levels in the healthy donors and joint-related diseases investigated in the two cohorts. (**A**) CALC2 levels of cohort 1. (**B**) CALC2 levels of cohort 2. Data are shown as mean ± SD, and CALC2 levels are compared using Mann–Whitney tests. Asterisks indicate the following: * *p* < 0.05, ** *p* < 0.01.

To assess the potential clinical relevance of the biomarker, CALC2 levels were measured in the serum samples from the two cohorts. The patients’ demographics are shown in Table 2 and Table 3. In cohort 1, the patients with RA and AS showed significantly lower CALC2 levels than the healthy donors (*p* = 0.01 and *p* = 0.02, respectively; Figure 5). No significant difference was observed between the patients with RA and AS. In cohort 2, no significant difference in CALC2 levels was found between the OA patients and the healthy donors.

## 3. Discussion

In this study, we developed a competitive chemiluminescence immunoassay (CLIA) for the detection of CALC2 using a monoclonal antibody targeting the N-terminal of PIICP. The main findings of the study were as follows: (1) the development of a specific and technically robust assay targeting the CALC2 sequence DQAAGGLRQH measuring the C-terminal pro-peptide of type II collagen; (2) CALC2 was generated by the BMP-1 cleavage of type II collagen; (3) the CALC2 level was increased in HEX supernatant in response to treatment with IGF-1, and the presence of CALC2 was further confirmed using Western blot analysis; (4) CALC2 levels were significantly lower in the patients with RA and AS than in the healthy controls.

In the field of rheumatology, simple and accurate non-invasive biomarkers for the assessment of disease activity and therapeutic efficacy in rheumatic diseases must be developed. The monoclonal antibody showed no cross-reactivity to either the elongated or truncated epitope, and its specificity was further confirmed using Western blot analysis. NBH-292 # 113 4B10-2B2-1D6-1D7 detected one band of approximately 35 kDa, which corresponds to the reduced molecule of PIICP, and a band of approximately 62 kDa, which corresponds to the unreduced molecule (i.e., interactions between polypeptides are preserved) [3]. To achieve a robust assay performance, key assay parameters such as buffer concentrations, assay incubation times, and temperatures were optimised.

Herein, we described a highly specific biomarker measuring the true formation of type II collagen. We showed in two separate ex vivo human cartilage OA explant experiments that IGF-1 treatment increased the type II collagen formation assessed using CALC2. In the pathogenesis of OA, cartilage changes are associated with excessive cartilage degradation [15]. Therefore, one treatment avenue being pursued is to increase cartilage production. In this setting, CALC2 might have the potential usefulness in the evaluation of the efficacy of anabolic treatment of articular cartilage in patients with OA, which remains challenging.

In healthy joint tissues, a finely tuned balance exists between the formation and degradation of type II collagen, maintaining physiological homeostasis [16]. However, excessive remodelling of type II collagen is a hallmark of rheumatic diseases. During the past decades, several biomarkers measuring cartilage formation have been developed by quantifying the N or C terminal of type II collagens, namely PRO-C2, NPII, PIIANP, Pcol II-C, PIICP, and CPII [6,10,11,12,13]. Biomarkers reflect the release of the N- and C-terminal pro-peptides of collagen type II. PRO-C2 measures the type IIB collagen N-pro-peptide splice variant of the N-terminal of type II collagen, and significantly lower levels were found in patients with OA than in healthy controls [6]. NPII also measures the N-terminal pro-peptide and was found to be significantly decreased in patients with OA compared with healthy donors [10]. PIIANP measures the type IIA collagen N pro-peptide splice variant and was found to be significantly decreased in patients with knee OA and RA [12]. Levels of the carboxy-terminal type II procollagen peptide, pCOL II-C, were significantly high in patients with OA and traumatic arthritis compared with patients with RA [11]. CPII measures type II collagen C-pro-peptide levels and was significantly elevated in the synovial fluid samples from patients with OA compared with patients with RA, but serum CPII levels were not increased [13]. These findings emphasise that different epitopes of type II collagen reflect different aspects of type II collagen turnover in rheumatic diseases. The CALC2 assay reflects the true formation of type II collagen, as the epitope is only detected after cleavage from matured collagen.

We showed that CALC2 levels were significantly lower in the patients with RA and AS than in the healthy donors. To our knowledge, this is the first study to show lower levels of type II collagen formation in patients with AS. In contrast to our results, Kim et al. [17] showed elevated CPII levels in patients with AS. However, in this study, the patients with AS had active disease; therefore, the rate of type II collagen formation in these patients is likely to change because of the increased tissue turnover.

The present study was exploratory; thus, the clinical studies used for validation included a relatively small population size. Only limited data from the cohorts were available. Therefore, other factors such as disease activity might have influenced the CALC2 levels. Further studies are needed in larger and better-characterised cohorts to validate the usefulness of CALC2 level as a diagnostic biomarker of rheumatic diseases. Lastly, chondrocalcin has been associated with calcification due to its calcium-binding properties. However, the role of CALC2 as a calcification marker was not investigated in this study.

## 4. Materials and Methods

### 4.1. Materials

The synthetic peptides were purchased from Genscript (Piscataway, NJ, USA; Table 4), and chemicals were purchased from Sigma-Aldrich (St. Louis, MO, USA) or Merck (Readington, NJ, USA), unless otherwise stated.

### 4.2. Immunogen Selection, Immunisation, and Fusion

The identification of chondrocalcin was based on a previous study [3]. The immunogen DQAAGGLRQH was conjugated to keyhole limpet hemocyanin (KLH). Five 6- to 7-week-old Balb/C female mice were immunised subcutaneously with 200 µL of emulsified antigen and 50 µg of immunogenic peptide (DQAAGGLRQH-GGC-KLH) using Stimune Immunogenic Adjuvant (SPECOL, Invitrogen, Carlsbad, CA, USA). The immunisations were performed at 3-week intervals until stable serum titre levels were reached. The mouse with the highest serum titre was rested for a month and then boosted intravenously with 50 μg of immunogenic peptide in a 100-μL 0.9% NaCl solution 3 days before isolation of the spleen for cell fusion. The mouse spleen cells were fused with SP2/0 myeloma cells to produce hybridoma cells, a method previously described by Gefter et al. [18]. The selected hybridoma clones were grown in 96-well microtiter plates using a standard limiting dilution method to secure monoclonal growth. Clones were continuously screened against the specific epitope, elongated peptide (ADQAAGGLRQH), truncated peptide (QAAGGLRQH), and nonsense peptide (KLH NBH-50). Two hybridoma clones (NBH292#33-1C6-2D2-2B4 and NBH-292#33 4B10-282-1D6-1D7) were selected on the basis of their high reactivity to the native samples. The immunoglobulins were purified from the supernatant using HiTrap Protein G HP affinity columns, in accordance with the manufacturer’s instructions (GE Healthcare Life Science, Buckinghamshire, UK). The antibody of one hybridoma clone (NBH-292#33 4B10-282-1D6-1D7) was chosen for further assay development and labelled with horseradish peroxidase (HRP) using a peroxidase labelling kit (Roche, Basel, Switzerland), following the manufacturer’s instructions.

### 4.3. Assay Protocol for CALC2 CLIA

To develop the CLIA, several settings were tested to select the reagents, their concentrations, incubation time, and temperature that worked best for the assay. The definite CALC2 CLIA protocol was as follows: A 96-well streptavidin-coated white microplate (Greiner Bio-One, Kremsmünster, Austria) was coated with 2.7-ng/mL biotinylated peptide (DQAAGGLRQH{LYS(Biotin)}) dissolved in assay buffer (25 mM PBS-BTB, 8 g NaCl, pH 7.4) and incubated for 30 min at 20 °C in the dark with 300 rpm shaking. Afterwards, 20 µL/well of standard peptide or sample was added to the appropriate wells, followed by 100 µL of 100-ng/mL HRP conjugated monoclonal antibody diluted in assay buffer and incubated for 20 h at 4 °C with shaking (300 rpm) in darkness. After each incubation step, the wells were washed with washing buffer (20 mM Tris, 50 mM NaCl, pH 7.2). The chemiluminescence substrate (BM Chemiluminescence Enzyme-linked Immunosorbent Assay [ELISA] substrate [POD], Roche, Basel, Switzerland) working solution was mixed 15 min before use, and 100 µL/well was added to the plate. The plate was analysed using Fluroskan FL 374-91652C (Thermo Scientific SkanIt, Thermo Fisher Scientific, Waltham, MA, USA) after 1 min of shaking (240 rpm) and a 2 min pause. A standard curve was generated by serial dilution of the selection peptide (50 ng/mL) and plotted using a 4-parameter logistic curve fit *Y* = (*A* − *D*)/(1 + (x/*C*)*^B^*) + *D*, where *R* > 0.9. Each plate included five kit controls to monitor inter-assay variation.

### 4.4. Technical Evaluation of CALC2

Technical assay validation was performed to test the assay stability and robustness. The intra- and inter-assay variations were determined by 10 independent runs, including eight quality controls and two internal control samples covering the detection range in double determinations. The lower limit of detection (LLOD) was established as the mean of 3 standard deviations (SD) from 21 determinations in blank samples (assay buffer). The lower limit of quantification (LLOQ) was calculated on the basis of five independent runs of four serum samples in three duplicates, and the upper limit of quantification (ULOQ) was based on 10 independent runs of the standard curve. IC50 (half-maximal inhibition concentration) was calculated from the standard curve. Dilution recovery was assessed from twofold dilutions of serum to calculate the linearity as recovery percentages of 100%, with the undiluted sample as reference value. To test the accuracy of the assay, peptide spiking recovery was determined. Here, different concentrations of the selection peptide were spiked in buffer and human serum to identify the matrix effect of the serum samples. The analyte stability was tested for three healthy human serum samples incubated at either 4 °C or 20 °C for 2, 4, 24, and 48 h. The recovery was calculated from the percentage variation from the sample kept at −20 °C (0 h sample). Furthermore, the analyte stability was assessed for three healthy human serum samples exposed to five freeze/thaw cycles, where the recovery was calculated from the sample that underwent only one freeze/thaw cycle. In addition, analytical interference was measured in healthy human serum spiked with low/high contents of haemoglobin (2.50/5 mg/mL), lipemia/lipids (1.50/5 mg/mL), and biotin (3/9 ng/mL). The interference was calculated as the percentage recovery of the analyte in non-spiked serum samples. All sample tests were run as double determinations.

### 4.5. Human Cartilage Explant Model

Two human cartilage explant model studies were investigated. The models included five and nine patients with OA, respectively, who underwent knee replacement surgery due to knee OA, and cartilage biopsies were isolated from the femoral condyles. Explants were excised using a 3 mm biopsy puncher, and the isolated explants were randomly distributed in 96-well culture plates with two explants per well, as described previously [19]. In the first study, explants were cultured with IGF-1 (100 ng/mL) for 35 days, and supernatant was collected on days 0, 7, 14, 21, 28, and 35 and stored at −20 °C for further biochemical analyses. In the second study, explants were cultured with IGF-1 (100 ng/mL) for 21 days, and supernatant was collected on days 0, 3, 5, 7, 12, 14, 19, and 21. The samples were pooled before CALC2 measurement at baseline (day 0) and in the early phase (days 3, 5, and 7), mid-phase (days 10, 12, and 14), and late phase (days 17, 19, and 21) because of lack of volume.

### 4.6. In Vitro Protease Cleavage

Recombinant human pro-collagen II (R&D Systems, Coppenhagen, Denmark) was incubated with BMP-1 (R&D Systems) and HEPES buffer (25 mM) for 24 h at 37 °C in a protein-to-protease ratio of 1:2. EDTA (5 mM) was added to stop the digestion reaction. All supernatants were stored at −80 °C until measurement in the CALC2 assay. The following samples were used as controls: digestion buffer alone, BMP-1, recombinant human pro-collagen II, mature human type II collagen (Chondrex, Redmond, WA, USA) incubated with BMP-1, or mature human type II collagen. The experiment was performed in three replicates.

### 4.7. Western Blot of HEX Supernatant

In the first HEX study, HEX supernatant obtained on days 0, 14, 21, 28, and 35 from one patient was electrophoresed on a NuPAGE 4-12% Bis-Tris gel (Invitrogen, Carlsbad, CA, USA) under reducing conditions using a NuPAGE MES SDS running buffer (Invitrogen). By using an iBlot Dry blotting system (Life Technologies, Carlsbad, CA, USA), the proteins from the polyacrylamide gel were transferred onto an iBlot nitrocellulose membrane (Life Technologies, Bengaluru, India). Thereafter, the membrane was blocked for 1 h with 5% skim milk (Sigma-Aldrich, St. Louis, MO, USA) in TBST (Tris-buffered saline [TBS] with 0.1%, Tween-20). The membrane was incubated overnight with monoclonal antibody NBH-292#33 4B10-282-1D6-1D7 at 4 °C. Next, the membrane was washed in TBST three times for 10 min and incubated in the secondary peroxidase conjugated antibody (1:5000) for 1 h. The membrane was washed in TBST, as previously described, and incubated for 1–5 min in SuperSignal west femto maximum sensitivity substrate (Thermo Fisher Scientific, Waltham, MA, USA). The bands were visualised on a C-DiGit Blot Scanner (LI-COR Biosciences, Lincoln, NE, USA).

### 4.8. Clinical Profiling of CALC2

CALC2 was measured in two cohorts obtained from the commercial vendor Proteogenex (Culver City, CA, USA) and Central BioHub (Hennigsdorf, Germany). Samples were collected after informed consent and approval by the local ethics committee were obtained and in compliance with the Declaration of Helsinki of 1975. Cohort 1 included patients with AS (*n* = 19), patients with RA (*n* = 18), and age- and sex-matched controls (*n* = 18). Cohort 2 included patients with OA (*n* = 8) and age- and sex-matched controls (*n* = 14). As an exploratory study, the inclusion criteria for participants were age and sex distributions.

### 4.9. Statistical Analysis

All data were treated as non-parametric. Differences between the treatments in the HEX studies were analysed using the Mann–Whitney test. Baseline differences between the groups of participants in cohorts 1 and 2 were examined using Kruskal–Wallis rank and Mann–Whitney tests. For all the statistical analyses performed, a *p* value < 0.05 was considered significant. Data analyses were performed using *R* studio version 4.0.3 (R Foundation for Statistical Computing, Vienna, Austria). Graphical illustrations were created using GraphPad Prism version 9.2.0 for Windows (La Jolla, CA, USA).

## 5. Conclusions

In conclusion, the CALC2 assay is a specific and technically robust CLIA assay for the detection of the C-terminal pro-peptide of type II collagen, which may be used for the quantification of type II collagen formation. This assay is a potential novel non-invasive serological biomarker for discriminating patients with RA and AS from healthy donors.

## Figures and Tables

**Table 1:** CALC2 assay technical validation summary (accepted recovery: 100% ± 20%).

**Assay Parameter**	**Result**
IC50	1.63 ng/mL
Measurement range (LLOQ–ULOQ)	0.44–44 ng/mL
Inter-assay variation *	12% (6.19–21.11%)
Intra-assay variation *	7% (4.91–11.47%)
Dilution recovery of human serum (*n* = 4) *	106% (102–113%)
Spiking recovery (serum in serum) (*n* = 3) *	83% (76–95%)
Analyte stability *	
2 h, 4 °C/20 °C 4 h, 4 °C/20 °C 24 h, 4 °C/20 °C 48 h, 4 °C/20 °C	98.9–113%/101–128.3% 95.6–110.5%/92.7–110.2% 100.6–115.8%/101.4–106.4% 98.5–101.5%/100.8–115.1%
Analyte recovery, 4 freeze/thaw cycles *	93.8% (62.4–113.8)
Haemoglobin recovery low/high *	109.6%/110.7%
Lipid recovery low/high *	99%/93%
Biotin recovery low/high *	93.1%/102.5%

* Percentages are reported as the mean of *n*, number of measurements. LLOQ, lower limit of quantification; ULOQ, upper limit of quantification.

**Table 2:** Demographics and CALC2 measurements from commercial cohort 1.

	Patients with RA (*n* = 18)	Patients with AS (*n* = 19)	Healthy Donors (*n* = 18)	*p* Value
Age, years	35.7 (3.3)	35.8 (3.2)	35.8 (3.8)	0.981
Sex, male	9 (50.0%)	10 (52.6%)	9 (50.0%)	0.983
BMI, kg/m^2^	24.1 (1.8)	22.9 (1.9) *	25.6 (3.0)	0.034
CALC2, ng/mL	0.6 (0.2)	0.6 (0.3)	1.2 (0.6)	<0.001

Categorical variables are presented as number (percentage); and continuous variables, as mean (standard deviation). The Kruskal–Wallis rank test was used to compare the differences among the groups; and Mann–Whitney tests, between each group and the healthy donors (the reference group). * *p* < 0.05, the patients with AS compared with the healthy donors. RA: rheumatoid arthritis; AS: ankylosing spondylitis; BMI: body mass index.

**Table 3:** Demographics and CALC2 measurements from commercial cohort 2.

	Patients with OA (*n* = 8)	Healthy Donors (*n* = 14)	*p* Value
Age, years	69.8 (4.1)	70.6 (1.2)	0.683
Sex, male	4 (50.0%)	7 (50.0%)	0.375
BMI, kg/m^2^	29.5 (3.0)	25.9 (2.0)	0.038
CALC2, ng/mL	0.6 (0.2)	0.8 (0.4)	0.729

Categorical variables are presented as number (percentage); and continuous variables, as mean (standard deviation). The Mann–Whitney test was used to compare the differences among the groups. OA: osteoarthritis.

**Table 4:** Amino acid sequences of the synthetic peptides used for monoclonal antibody production and CLIA validation.

Peptide Type	Sequence
Immunogenic peptide	DQAAGGLRQH-GGC-KLH *
Standard peptide	DQAAGGLRQH
Screening peptide	DQAAGGLRQH{LYS(Biotin)}
Elongated peptide	ADQAAGGLRQH
Truncated peptide	QAAGGLRQH
Non-sense coating peptide	MSAFAGLGPRK-Biotin
Non-sense standard peptide	MSAFAGLGPR

* Keyhole limpet hemocyanin.

## Data Availability

The data presented in this study are available upon reasonable request from the corresponding author.
